# Felodipine re-positioned as a neuroprotectant via improved optic nerve head blood circulation in retinal ischemic rabbits and ocular hypertensive rats

**DOI:** 10.1038/s41598-025-09733-1

**Published:** 2025-07-03

**Authors:** Takazumi Taniguchi, Takahiro Akaishi, Tatsuya Hata, Rieko Yamashita, Yoshihiko Esaki, Ryo Morofuji, Komei Okabe, Masahiro Fuwa, Yasuko Yamamoto, Li Guo, Soyoung Choi, Vy Luong, Takashi Ota, Najam A. Sharif, M. Francesca Cordeiro, Masaaki Kageyama

**Affiliations:** 1 Ophthalmology Innovation Center, Santen Pharmaceutical Co., Ltd, 8916-16 Takayama-cho, Ikoma-shi, 630 − 0101 Nara Japan; 2 Product Development Division, Santen Pharmaceutical Co., Ltd, Ikoma-shi, Nara Japan; 3 Institute of Ophthalmology, University College London (UCL), London, UK; 4 Novai Ltd, Work Life, The White Building, 33 King’s Road, Reading, RG1 3AR UK; 5 Imperial College of Science and Technology, St. Mary’s Campus, London, UK; 6 Ophthalmology Innovation Center, Santen Inc, Emeryville, CA USA; 7 Eye-APC Duke-NUS Medical School, Singapore, Singapore; 8 Department of Pharmacy Sciences, Creighton University, Omaha, NE USA; 9 Department of Pharmaceutical Sciences, College of Pharmacy and Health Sciences, Texas Southern University, Houston, TX USA; 10 Department of Pharmacology and Neuroscience, University of North Texas Health Sciences Center, Fort Worth, TX USA; 11 Singapore Eye Research Institute, Singapore, 169856 Singapore

**Keywords:** Glaucoma, Calcium channel blocker, Felodipine, Retinal ganglion cells, Ocular blood flow, Neuroprotection, Glaucoma, Optic nerve diseases

## Abstract

The vasodilatory and neuroprotective properties of felodipine were characterized in normal and ischemic rabbit eyes, an ocular hypertension (OHT) rat model, and cultured human neuron-like cells. Blood flow in the optic nerve head (ONH) was measured using laser speckle flowgraphy following intravitreally injected felodipine into normal and ischemic rabbit eyes receiving endothelin-1 injection. Felodipine concentrations in the retina-choroidal tissue were determined using mass spectrometry, and ocular safety was assessed using in-life examinations and histopathology in rabbits. The number of retinal ganglion cells (RGCs) was counted following intravitreal felodipine injection in hypertonic saline-induced OHT rats. The in vitro neuroprotective effects of felodipine against vincristine-induced nuclear loss and neurite shortening were evaluated in differentiated SH-SY5Y neuron-like cells. Felodipine increased ONH blood flow in a dose-dependent manner in normal rabbit eyes. High felodipine dosage (780 nmol/eye) improved blood circulation in endothelin-1-induced ischemic eyes. The ONH blood flow response to felodipine correlated with tissue concentration-time profiles. Even high dose of felodipine did not cause any ocular toxicity. Furthermore, felodipine protected SH-SY5Y cells against vincristine-induced damage and prevented OHT-induced RGC cell loss following its administration (40 nmol/eye). Felodipine can act as a neuroprotectant against glaucomatous neuropathy via improved ONH microcirculation and direct neuroprotection of RGCs.

**Supplementary Information:**

The online version contains supplementary material available at 10.1038/s41598-025-09733-1.

## Introduction

Glaucoma is a leading cause of irreversible vision impairment and blindness worldwide^1,2^. It is a form of optic neuropathy characterized by optic nerve damage and retinal ganglion cell (RGC) death, resulting in progressive visual field defects^1,3^. The most common form of glaucoma is primary open angle glaucoma, which includes normal tension glaucoma. Since elevated intraocular pressure (IOP) is the most validated risk factor for glaucomatous optic neuropathy, medical and surgical IOP-lowering therapies remain the standard of care for this eye disease^1,3^. Despite robust IOP management, visual field defects continue to deteriorate in a relatively high proportion of patients with glaucoma, suggesting that IOP-independent mechanisms underlie optic nerve damage and/or RGC death^3–6^. Although efforts have focused on addressing this issue, direct neuroprotective approaches using small-molecule drugs, cells, or gene therapies remain largely unsuccessful^3–6^.

Among all the proposed mechanisms, such as mechanically impaired neurotrophic support, oxidative stress, and immune dysfunction^3–6^, the vascular theory seems to be one of the most compelling hypotheses, in which abnormalities in the optic nerve head (ONH) microcirculation may play a substantial role in glaucomatous neurodegeneration and development of glaucomatous optic neuropathy (GON)^7,8^. Impaired and reduced blood supply to the ONH may damage the optic nerve fibers and RGC somata via ischemic neuroinflammatory and neurodegenerative mechanisms. To validate this hypothesis, many investigators have tested general vasodilators including calcium channel blockers, endothelin receptor antagonists, and nitric oxide donors in animal models of GON^9,10^. In particular, various calcium channel blockers have been widely studied for their potential to improve blood circulation in the ONH, thereby preventing glaucomatous neurodegeneration^9^. In animal studies, calcium channel blockers such as lomerizine, nimodipine, and nifedipine have been shown to protect RGCs, probably through increased blood flow in the optic nerve and/or direct neuroprotection^9,11,12^. Consistently, one clinical study demonstrated that oral nilvadipine increases ONH and posterior choroidal circulation with slowed visual field deterioration in patients with glaucoma^13^ and several studies using nimodipine reported the beneficial effects on visual function^9^. On the other hand, the findings of other clinical studies contradict these observations^9,14–16^. For examples, previous studies did not consistently agree on the effects of nifedipine on visual field outcomes^9^. Thus, these earlier findings warrant further studies to test the potential of other calcium channel blockers as neuroprotective agents in the treatment of glaucoma/GON.

Felodipine is a selective L-type calcium channel blocker that has been used as an antihypertensive drug in clinical settings for many years^17,18^. Interestingly, recent studies have demonstrated that felodipine can be re-positioned as a neuroprotectant, inducing autophagy in the mouse brain and removing undesired misfolded proteins associated with multiple neurodegenerative diseases^19^. In addition, the prevention of neurite shortening caused by antiviral and antitumor drugs were observed in cultured mouse dorsal root ganglia neurons treated with felodipine^20^. Furthermore, a felodipine film used for ocular delivery lowered IOP in normotensive rabbits and suppressed carrageenan-induced inflammation in rabbits^21^. Thus, we hypothesized that felodipine might possess unique pharmacological characteristics, namely vasodilatory, direct neuroprotective, and anti-inflammatory properties, and be an ideal neuroprotective candidate for the treatment of glaucoma, particularly in patients refractory to the standard of care.

The present study was designed to characterize the vasodilatory effects of intravitreally injected felodipine in the rabbit ONH, along with its direct neuroprotective effects in differentiated human SH-SY5Y cells and in ocular hypertensive rats. Here, we report that intravitreally administered felodipine reaches pharmacologically active concentrations in the retinal choroid, leading to improved ONH blood flow and neuroprotection of RGCs.

## Results

### Improved ONH blood flow following intravitreal injection of felodipine in rabbits under normal and ischemic conditions

**Fig. 1:**
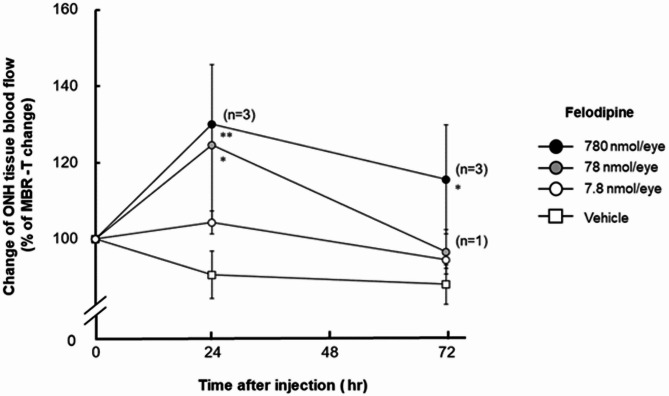
Effects of intravitreal injection of felodipine on tissue blood flow (MBR-T) in the optic nerve head (ONH) in conscious rabbits. Vehicle or felodipine (7.8, 78, and 780 nmol/eye) was injected into the vitreous of the left eye of each animal, whereas the right eye remained untreated. ONH tissue blood flow was determined using laser speckle flowgraphy before injection, and 24 and 72 h after injection. ONH blood flow was expressed as a percentage of the pretreatment value (baseline). Each value represents the mean ± SEM of 1 to 4 eyes. Statistically significant differences are shown as * *P* < 0.05, ** *P* < 0.01, compared to the vehicle group at the respective timepoints (Dunnett’s multiple comparison test).

**Fig. 2:**
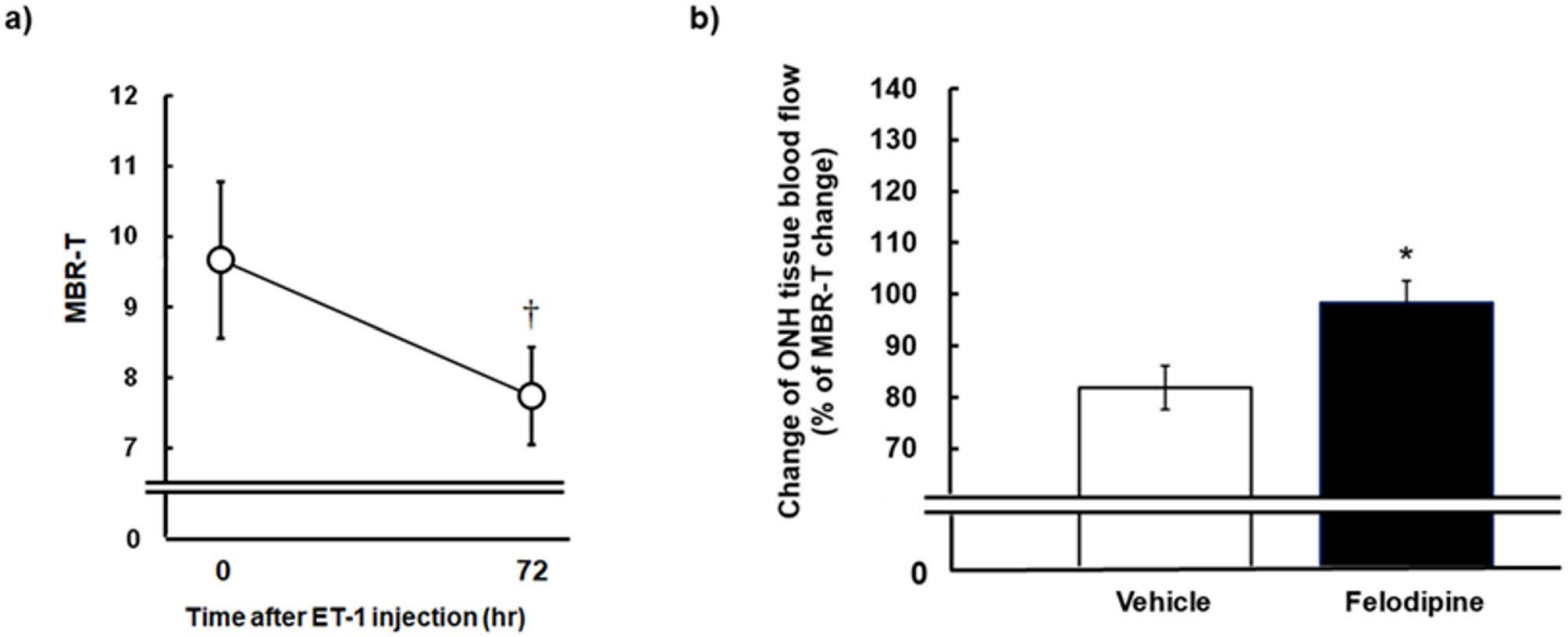
Preventive effects of intravitreal injection of felodipine against impaired optic nerve head (ONH) blood flow induced by endothelin-1 (ET-1) in the rabbit eyes. Under a conscious condition, each animal received intravitreal injection of vehicle or 780 nmol/eye felodipine prior to ET-1 (5 pmol/eye) intravitreal injection. ONH tissue blood flow was measured by laser speckle flowgraphy before and 72 h after ET-1 injection. (**a**) Effect of ET-1 alone on tissue blood flow (MBR-T) measured before and 72 h after concurrent injection of ET-1 and vehicle. ^†^*P* < 0.05 (Paired t-test) compared with the baseline (time 0). (**b**) Effect of vehicle or felodipine on ET-1-induced ONH blood flow impairment. ONH blood flow measured at 72 h after ET-1 injection is expressed as a percentage of the pre-treatment value (baseline) in each group. *P < 0.05 (Student’s *t*-test) compared with vehicle. Each value represents the mean ± SEM of 7 to 8 eyes.

We first examined the effects of felodipine on tissue blood flow in the ONH of conscious, normal rabbit eyes, and ischemic eyes receiving endothelin-1. As shown in Fig. 1, a single injection of felodipine (7.8, 78, and 780 nmol/eye) into normal rabbit eyes increased ONH blood flow in a dose-dependent manner, and its effect peaked 24 h after administration. The peak levels at the middle and highest doses reached statistical significance compared to the vehicle-injected control (*P* = 0.0119 and 0.0076 at doses of 78 nmol/eye and 780 nmol/eye, respectively). The maximum increase in ONH blood flow at the highest dose was approximately 30% from baseline and remained statistically significant even at 72 h post administration (*P* = 0.0314). As reported in our previous study^22^, a single intravitreal injection of endothelin-1 (5 pmol/eye) moderately but significantly reduced ONH blood flow in rabbit eyes via vasoconstriction of retinal blood vessels (Fig. 2a). A single intravitreal administration of felodipine prior to endothelin-1 treatment significantly suppressed endothelin-1-induced ONH blood flow impairment compared with the vehicle control (Fig. 2b, *P* = 0.0218).

**Fig. 3:**
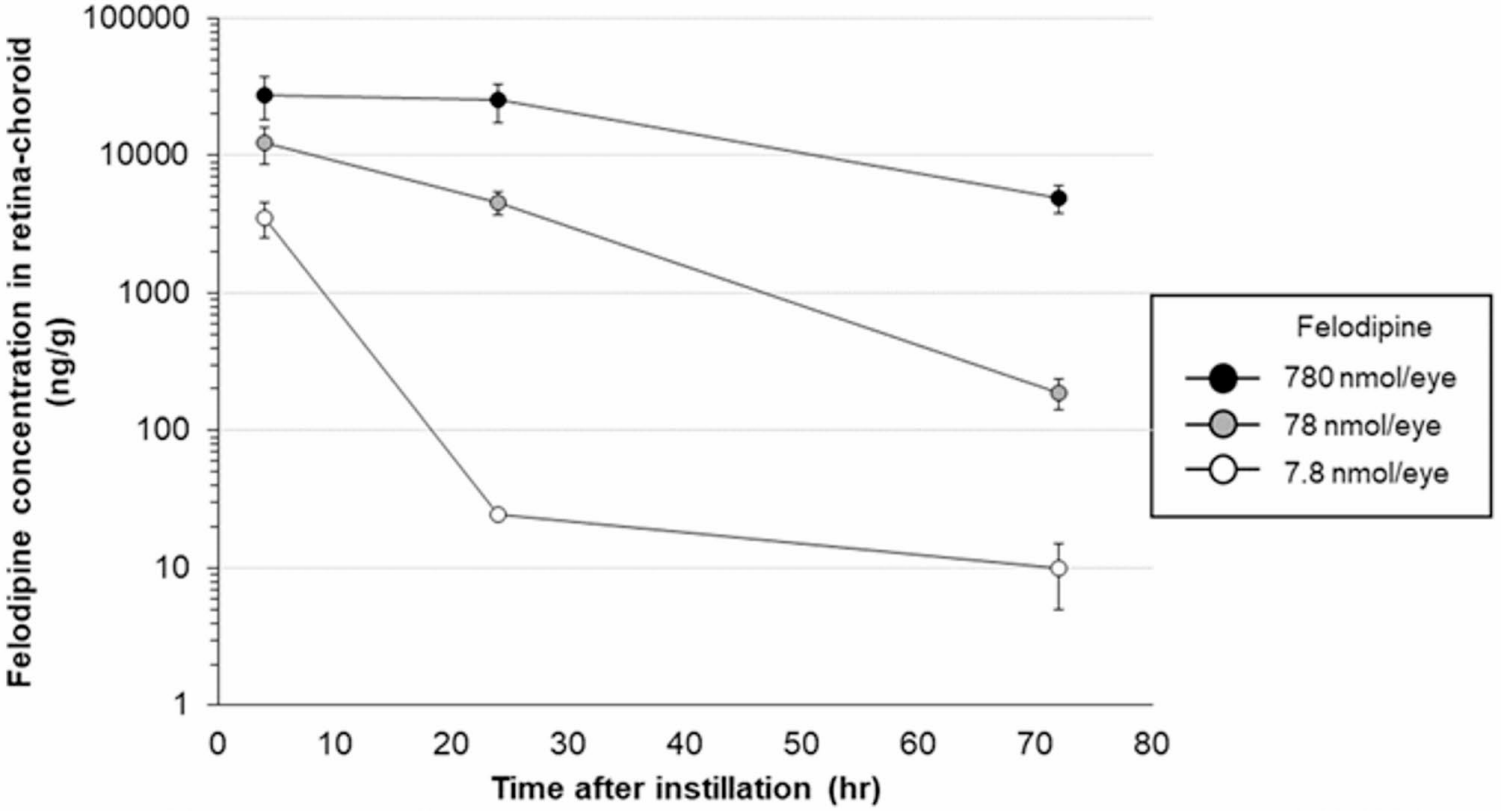
Tissue felodipine concentration-time profiles in the retina-choroid of the rabbit eyes receiving intravitreal injection of felodipine (7.8, 78, and 780 nmol/eye). Eyes were enucleated 4, 24, and 72 h following its injection. The retina-choroid were isolated and tissue felodipine concentrations were determined using mass spectrometry. Each value represents the mean ± SEM (*n* = 4–6 eyes).

Next, we measured the concentrations of felodipine in the rabbit retina-choroid tissue following intravitreal injection of the same three doses (Fig. 3). The felodipine levels in the retina-choroid tissues increased in a dose-dependent manner and reached a peak level 4 h following injection. The duration of increased felodipine concentrations was also dose-dependent and remained at pharmacologically active levels^18^ even 72 h after injection of the highest dose. Using slit-lamp biomicroscopy and indirect ophthalmoscopy, we confirmed that felodipine (7.8, 78, and 780 nmol/eye) did not cause any signs of toxicity/damage in any ocular tissue on days 1, 2, and 7 after injection (Table 1). Consistently, histopathological examination revealed no toxic ocular effects of the intravitreal injection of felodipine on day 8 (Table 1).

**Table 1:** In-life and histopathological assessments of toxicity following single intravitreal injection of felodipine in rabbits.

Group		Vehicle		Felodipine (7.8 nmol/eye)		Felodipine (78 nmol/eye)		Felodipine (780 nmol/eye)	
Animals (eyes)		3 animals (6 eyes)		3 animals (6 eyes)		3 animals (6 eyes)		3 animals (6 eyes)	
Clinical signs		-		-		1 to 6 h ^a)^: Lacrimation 1/3, right eye		-	
Body weight		-		-		-		-	
Slit lamp examination	Eye	Right	Left	Right	Left	Right	Left	Right	Left
	Day 1–4 h ^a)^	-	-	-	PCO⁑1/3	Floater# 3/3	Floater# 1/3	TA 3/3	TA 3/3
	Day 2	-	-	-	PCO⁑1/3	-	-	TA 2/3	TA 3/3
	Day 7	-	-	-	PCO⁑1/3	-	-	TA 3/3	TA 3/3
Fundus examination	Day 1–4 h ^a)^	-	-	-	-	-	-	-	-
	Day 2	-	-	-	-	-	-	-	-
	Day 7	-	-	-	-	-	-	-	-
Histopathology	Granuloma/injection site ^b)^	0/1^c)^		0/3 ^c)^		0/1 ^c)^		±: 1/1^c)^	

TA: Test article; PCO: Posterior capsular opacity. ±: Very slight, -: No specific findings. #Diffused drug particles ⁑: caused by intravitreal injection procedure. (a) Time after intravitreal injection; (b) foreign body reaction; (c) number of findings of granuloma/injection site on the eyeballs in which the location of the administration needle puncture could be seen on the section slide. The lacrimation was unrelated to the drug. The floaters were not toxic because they appeared to be aggregated drug particles that diffused from a depot of the injected test article during the in-life examination. Granuloma at the injection sites was a foreign body reaction but was not considered to be drug-related toxicity.

### Protective effects of felodipine against vincristine-induced cellular damage in differentiated SH-SY5Y cells

**Fig. 4:**
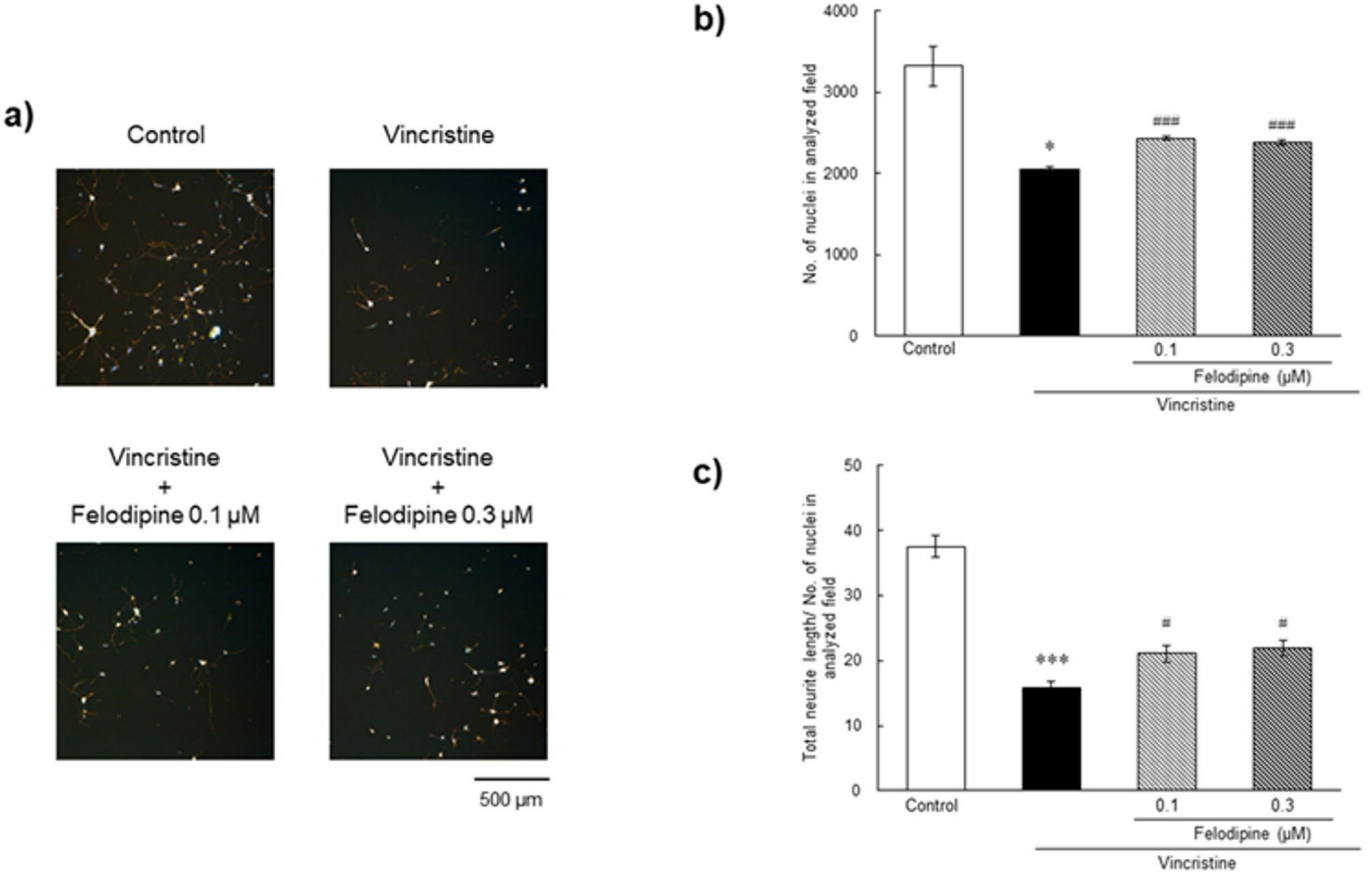
Neuroprotective effects of felodipine against vincristine-induced cellular damage in differentiated SH-SY5Y cells. The nucleus and neurites were stained with the cell membrane stain kit and hoechst33342 following treatment with vehicle (control, 0.1% DMSO), vincristine alone (200 nM), or felodipine (0.1 and 0.3 µM) plus vincristine. (**a**) Representative images of nuclear (blue) and neurite (orange) staining with or without drug treatments. The scale bar shows 500 μm. (**b**,**c**) Quantitative data to assess neuroprotective effects of felodipine. The number of nucleus and total neurite length per number of the nucleus were quantified in each analyzed field. Each value represents the mean ± SEM of 3 wells. * *P* < 0.05 *** *P* < 0.001, compared with the control group by either Student’s *t*-test or Aspin-Welch test. ^#^ *P* < 0.05, ^###^ *P* < 0.001, compared with vincristine alone by Dunnett’s multiple comparison test.

We speculated whether felodipine could confer neuroprotection indirectly via improved ONH blood flow and/or by directly acting on dying neurons. To address this question, we first examined if felodipine might elicit direct neuroprotective effects in cultured human neuron-like cells, namely differentiated SH-SY5Y cells. Figure 4 shows representative images of cell morphology and quantitative results of cell survival in the control (0.1% DMSO), vincristine treatment alone, and felodipine plus vincristine treatment groups. In the control group, the differentiated SH-SY5Y cells exhibited well-developed and interconnected neurites between the cell somata of neighboring cells (Fig. 4a). Felodipine alone (0.1 and 0.3 µM) did not cause any morphological changes in these cells following a 24-h treatment (Supplementary Fig. S1). Treatment with vincristine for 24 h remarkably reduced the number of cell nuclei (Fig. 4a and b) and shortened the total neurite length of the SH-SY5Y cells (Fig. 4a and c). When treated concurrently with vincristine, felodipine at both tested concentrations moderately suppressed the neurotoxic effects of vincristine, and these protective effects were statistically significant compared with those of vincristine treatment alone (*P* = 0.0001 and 0.0003 at 0.1 µM and 0.3 µM, respectively, in the number of cell nuclei, and *P* = 0.0326 and 0.0173 at 0.1 µM and 0.3 µM, respectively, in the total neurite length). These results suggest that felodipine exerts a direct neuroprotective effect against vincristine-induced damage in human neuron-like cells in vitro.

### Rescue effects of intravitreal felodipine on RGCs against ocular hypertensive injury in rats

**Fig. 5:**
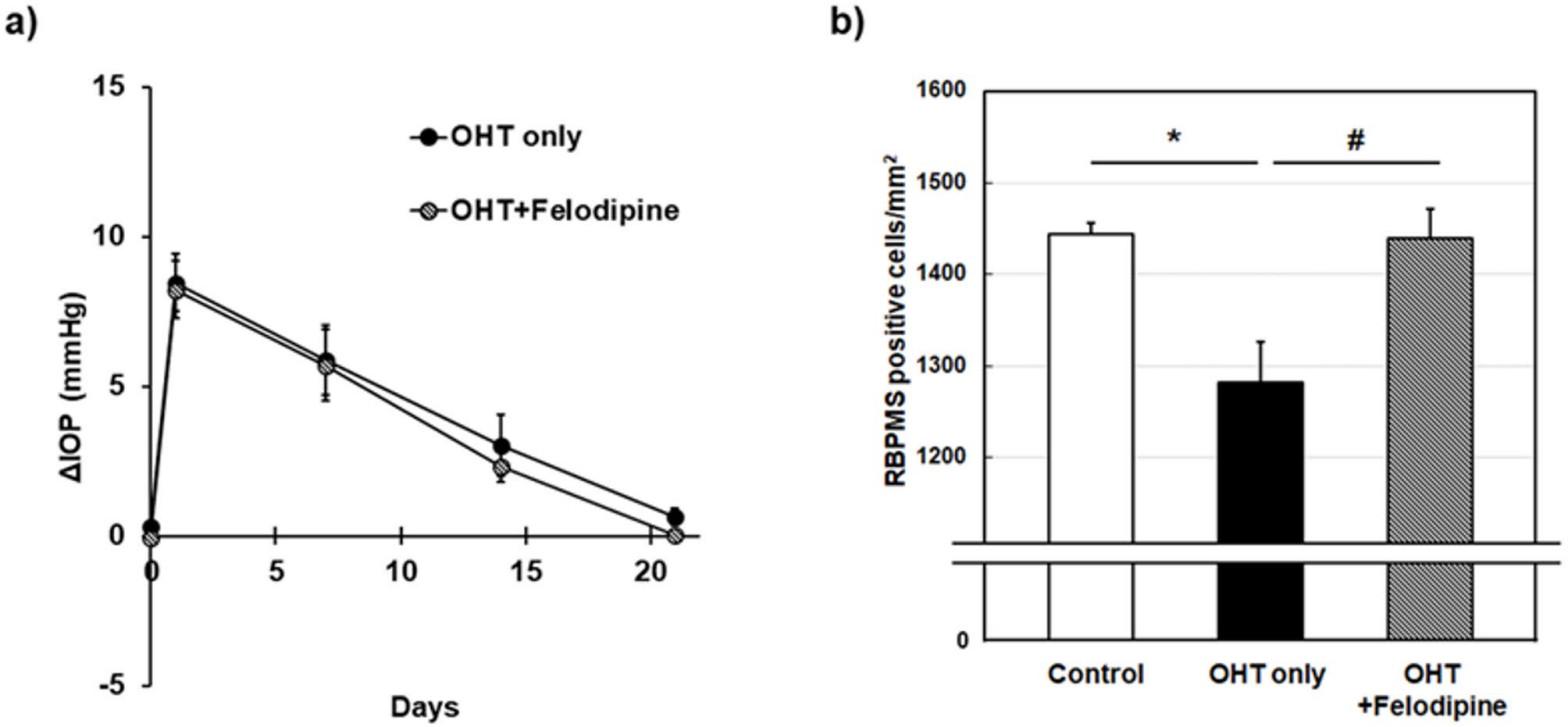
Protective effects of intravitreal injection of felodipine against ocular hypertension (OHT)-induced retinal ganglion cell damage in rats. (**a**) OHT was induced by episcleral venous injection of hypertonic saline into the left eye of each animal and the contralateral eye remained untreated. Felodipine (40 nmol/eye) was intravitreally injected twice on the day of OHT surgery and 11 days later. The IOP profiles were presented as the difference between the OHT eyes and the contralateral eyes. Each value represents the mean ± SEM of 5 eyes. (**b**) Determination of retinal ganglion cells (RGCs) density based on the staining of RNA-binding protein with multiple splicing (RBPMS) in OHT rats. Eyes were enucleated from normal (control), OHT without drug treatment (OHT only) and OHT rats receiving intravitreally injected felodipine (OHT + felodipine) three weeks following OHT surgery. RGCs were detected by immunostaining using an anti-RBPMS antibody on whole-mounted retinas. Each value represents the mean ± SEM of 5–6 eyes. * *P* < 0.05, compared with the control group by Aspin-Welch test; ^#^ *P* < 0.05, compared with the OHT only group by Student’s *t*-test.

Finally, we examined if felodipine might prevent high IOP-induced RGC loss independent of IOP changes following intravitreal injection in a rat model of ocular hypertension (OHT). The baseline IOP before the induction of OHT was 10.35 ± 0.14, and 10.14 ± 0.13 mmHg in OHT without drug treatment (OHT only) and OHT plus felodipine treatment groups, respectively. As shown in Fig. 5a, IOP rapidly increased and reached peak levels on day 1 in response to the hypertonic saline injection. Subsequently, it gradually declined over the remaining study period and returned to baseline levels by day 21 in both groups. Intravitreal injections of felodipine (40 nmol/eye) did not alter the temporal patterns of the OHT, suggesting that felodipine has no IOP-lowering effects in this setting in this species. OHT significantly reduced the density of RNA-binding protein with multiple splicing (RBPMS)-positive RGCs (*P* = 0.0189) compared to the control group (Fig. 5b). Intravitreal injection of felodipine completely prevented OHT-induced RGC loss. The RBPMS-positive RGC counts were significantly different from those observed in the OHT-only group (*P* = 0.0204) and were comparable to those in the control group, supporting the IOP-independent neuroprotective potential of felodipine.

## Discussion

The major finding of the present study was that the intravitreal injection of felodipine, a calcium channel blocker, remarkably improved ONH blood flow without causing ocular toxicity after reaching pharmacologically active levels in the retinal choroid. We also found that this compound exhibited significant neuroprotective effects against OHT-induced RGC loss in rats and vincristine-induced cell toxicity in cultured human neuron-like cells. These results suggest that intravitreal felodipine can be re-positioned as a potential direct and/or indirect neuroprotectant by improving the ONH blood circulation.

The results of previous studies on the neuroprotective effects of various calcium channel blockers through improved ONH blood circulation in animals and humans remain controversial. For example, oral administration of nilvadipine, nimodipine, and lomerizine has been reported to increase ocular blood flow and/or provide beneficial effects on the visual fields in patients with glaucoma and protect RGCs in experimental animal models^9,13^. In contrast, several other studies have failed to confirm the positive results of calcium channel blockers in patients or animal models^9^. In the present study, we found that intravitreally injected felodipine significantly improved ONH blood circulation in normal and ischemic rabbit eyes, protected RGCs against OHT-induced cell death in rats, and chemically induced cell death in differentiated SH-SY5Y cells. Complex factors related to experimental conditions, such as dosage, route of administration, and selection of compounds, may explain these inconsistent results across various studies. However, to our knowledge, this is the first report of the ocular vasodilatory and neuroprotective properties of felodipine in glaucomatous RGC injury.

To translate our findings from bench to clinic, it is crucial to ensure that there is a strong correlation between pharmacodynamic and pharmacokinetic parameters, at least in animal studies. Notably, our study demonstrated that the temporal patterns of improved ONH blood flow and retinal-choroidal felodipine concentrations were similar following intravitreal injection, suggesting good concordance between these two parameters. Considering these pharmacodynamic and pharmacokinetic characteristics, we estimated the minimum effective tissue levels of felodipine to be approximately 4.6 µg/g (corresponding to 11.9 µM at the molecular weight of 384.25), which was the level observed with the middle dose at 24 h. Additionally, the vitreous felodipine concentrations were estimated to be approximately 250 µg/mL (corresponding to 650 µM) to exhibit RGC protection in the rat OHT model (15 µg felodipine/eye and 60 µL vitreous volume). In our in vitro study, the effective concentrations of felodipine were 0.1 and 0.3 µM to prevent vincristine-induced neurotoxicity. Clearly, the in vitro tested concentrations of felodipine and those achieved in target tissues in vivo are significantly greater than the concentration needed to achieve half maximal blood vessel relaxation (IC_50_ = 0.15 nM) in coronary artery^18^. However, surprisingly, these estimated concentrations were much higher than the blood levels achieved following oral felodipine in clinical use (2–20 nM)^17^. Although the exact mechanism for this discrepancy is unknown, the regional sensitivity of blood vessels to felodipine may determine the necessary drug levels for vasodilation in a given tissue^23^. Interestingly, an earlier human study failed to show the efficacy of oral administration of felodipine on ocular blood flow in non-glaucoma volunteers, even though it lowered systemic blood pressure^24^. This finding could be explained by the possibility that oral administration may not deliver a sufficient amount of felodipine into the retina. Thus, the local ocular injection (and/or even topical ocular dosing) of felodipine may be an ideal route of administration to achieve much higher retinal-choroidal tissue concentrations, leading to clinical efficacy in ONH blood flow in humans.

Previous research indicated that felodipine exhibited greater potency in inducing autophagy than other tested calcium channel blockers (CCBs) in primary neuronal cells and exerted neuroprotective effects in mouse models of neurodegenerative diseases^19^. These findings suggest that felodipine has unique pharmacological characteristics among CCBs, making it a promising candidate for evaluation in glaucomatous models. Felodipine was also reported to lower IOP following the ocular application of a film designed for the sustained release of this drug in rabbits^21^. Conversely, felodipine did not alter IOP in OHT rats in the present study. We also observed no IOP reduction in normotensive rabbits following the intracameral injection of felodipine in a separate study. Therefore, this discrepancy may not simply be due to a species difference but may stem from differences in formulations (film vs. injectable solution) and/or the route of administration (subconjunctival installation vs. intravitreal injection). Regardless of this discrepancy, we believe that the improved ONH blood flow and RGC protection in response to intravitreal felodipine injections may be independent of IOP in our study. The question then arises as to whether the felodipine-induced RGC protection may be mediated indirectly by improved ONH blood flow or by direct action on RGCs. Several lines of evidence indicate that primary open-angle glaucoma is associated with impaired ONH blood circulation in both high IOP and normal-tension glaucoma (NTG)^7–10^. Interestingly, an earlier study demonstrated that plasma endothelin-1 (ET-1) levels were elevated in patients with glaucoma and may be associated with progressive neuropathy^25^. In animal studies, ET-1 has often been used to induce optic nerve ischemia and to recapitulate the clinical features of GON^22,26–28^. In the present study, felodipine significantly increased ONH blood flow in both normal and ET-1-induced ischemic rabbit eyes. Although we did not measure ONH blood flow in the OHT rat model, it is reasonable to conclude that felodipine may confer direct RGC protection and/or indirect protection via improved ONH blood circulation in vivo. We observed that felodipine protected human neuron-like cells against chemically induced cell death in vitro. Further studies are underway to clarify the relative contributions of indirect and direct components to the protective effects of RGCs observed in vivo.

In addition to its pharmacological properties, we characterized the safety profile of intravitreal felodipine in rabbits and demonstrated no specific safety concerns in the ocular tissues of the animals. However, the critical issue still remains unresolved as to the duration of action of felodipine in vivo as a neuroprotectant for human use in glaucoma. Our study found that, following felodipine injection at the highest dose, the increased ocular blood flow lasted for at least 72 h. This implies that patients may need to receive repeated intravitreal injections within a short period given the chronic nature of the disease. To solve this issue, the ocular use of felodipine may require a sustained-release formulation such as a rod shape implant, in situ depot or via minimally invasive devices. However, this approach is extremely challenging because of its water-soluble nature in aqueous solution, which limits its vitreous retention time. Nevertheless, the unique and favorable pharmacological properties of felodipine as a neuroprotectant with improved ONH microcirculation warrant further studies to develop its sustained-release form (e.g., at least a 3-month dosing interval).

In summary, the present study demonstrates that intravitreal injection of felodipine improves ONH blood circulation in normal and ischemic rabbit eyes and protects RGCs and human neuron-like cells against mechanically and chemically induced damage. These findings strongly support the notion that intravitreal injection of felodipine may be a potential therapeutic option, particularly for patients with progressive optic neuropathy and even those with well-controlled IOP. Further studies are underway to better characterize the non-clinical properties of felodipine and develop a novel formulation for its sustained release over several months.

## Methods

### Preparation of drug suspension

Felodipine was purchased from Tokyo Chemical Industry Co., Ltd. (Tokyo, Japan) and BLD Pharmatech Ltd. (Shanghai, China) for use in the rabbit and rat experiments, respectively. This compound was micronized and suspended in a solution containing buffering and stabilizing agents as well as a dispersant, and its tonicity and pH (pH 7.0) were adjusted. Solutions containing different amounts of felodipine were prepared for intravitreal administration in rabbits (0.1, 1, and 10 mg/mL, or 0.26, 2.6, and 26 mM, respectively) and rats (3.8 mg/mL or 9.88 mM).

### Animals

All the animals were treated in compliance with the ARVO statement for the Use of Animals in Ophthalmic and Vision Research. We also complied with the Basic Policies for the Conduct of Animal Experiments in Research Institutions” issued by the Ministry of Health, Labor, and Welfare, Japan (2006), and “The Guidelines for Proper Conduct of Animal Experiments” published by the Science Council of Japan (2006). The experiments using rabbits were approved and monitored by the Institutional Animal Care and Use Committee of Santen Pharmaceutical. The use of rats was approved by the local ethics committee at the University College London Institute of Ophthalmology and adhered to the United Kingdom Home Office regulations for the care and use of laboratory animals, the United Kingdom Animals (Scientific Procedures) Act (1986). Every effort was made to avoid the unnecessary use of laboratory animals. All animal experiments were carried out following ARRIVE guideline. For rabbit and rat studies, male Japanese White rabbits (Kitayama Labes) weighing 2.0 to 3.6 kg and male Dark Agouti (DA) rats (Janvier Labs) aged 8–10 weeks were used.

### ONH blood flow measurements in the normal and ischemic rabbit eyes

The method used to measure tissue blood flow has been described in detail previously^29^. Briefly, rabbits were randomly assigned to each group once they were enrolled in the study, and they were kept in a holding box and given a drop of 0.4% tropicamide (Mydrin M^®^; Santen Pharmaceutical Co., Ltd., Osaka, Japan) onto their left eyes for pupil dilatation. The sample size was determined based on previous in-house preliminary studies. The tissue blood flow in the ONH was determined using laser speckle flowgraphy (LSFG-NAVI) (Softcare, Fukuoka, Japan). The mean blur rate (MBR) in the optic disc was measured, and MBR-T values, that is, the average MBR in the optic disc area subtracted by that in the vessel area, were calculated using version 3.2.19.0 of the LSFG Analyzer, as the index of ONH blood flow. Following baseline measurements, the eyes were anesthetized locally with 0.4% oxybuprocaine hydrochloride (Benoxil 0.4% solution, Santen Pharmaceutical Co., Ltd., Osaka, Japan). Vehicle or felodipine suspension (30 µL/eye, 7.8, 78, and 780 nmol/eye) was injected into the posterior vitreous of the left eye of each animal via the pars plana through a Hamilton syringe with a 27-gauge needle and the fellow eye remained untreated. The MBR and MBR-T were determined again in the same area as the baseline. At each time point, MBR-T values were obtained five times consecutively and averaged as representative values. When the images for MBR-T value calculation were not acquired properly, the eyes were excluded from the study. Optic nerve ischemia was induced by intravitreal endothelin-1 injection (5 pmol/eye or 250 nM, 20 µL, Peptide Institute, Osaka, Japan) as described previously^22^. Endothelin-1 was injected 5 min after felodipine administration (780 nmol/eye) in the same manner as described above.

In separate experiments, felodipine tissue concentrations were measured in rabbits. Before drug administration, ophthalmological examinations were conducted on the rabbits. The animals observed with abnormalities were excluded, and the remaining animals were randomly assigned to each group. The sample size was determined based on previous in-house preliminary studies. Felodipine suspension (7.8, 78, and 780 nmol/eye) was intravitreally injected into the left eye of each rabbit as described above. At 4, 24, and 72 h after felodipine injection, eyes were enucleated from each animal receiving intravenous injection of an excess amount of pentobarbital sodium, immediately frozen on dry ice and stored at − 80 °C. On the day of the assay, individual retina-choroid tissues, including those near the optic nerve head area, were homogenized in water and deproteinized with acetonitrile. An aliquot of a homogenized sample (50 µL per each eye) was taken and mixed with 5 µL of nisoldipine solution as an internal standard (100 ng/mL). A resultant sample (10 µL) was analyzed using a QTRAP6500 + tandem mass spectrometer connected with a Shimadzu Nexera HPLC (Shimadzu, Japan) and an electrospray ionization source (Sciex, Tokyo, Japan). For a mobile phase, 0.02% formic acid and 0.02% formic acid in acetonitrile were mixed at a 1 to 1 ratio and pumped at a rate of 0.4 mL/min (isocratic elution). The mass spectrometer was operated in positive ion mode using multiple reaction monitoring, and the following transitions were recorded: felodipine m/z [M + H] 384.1/351.8, and IS (nisoldipine) m/z [M + H] 389.1/357.0. A calibration curve for the retinal-choroidal felodipine concentrations was generated to cover the range of 0.05–50 ng/mL. Data acquisition and quantitative analysis were performed using Analyst 1.6.2 software (Sciex, Tokyo, Japan).

For safety, the rabbits were randomly assigned to each group once they were enrolled in the study. The rabbits were excluded from the study if abnormalities were observed in clinical sign observation, body weight measurement, or ophthalmological examination performed before group allocation. From the perspective of confirming dose-dependency and considering individual variations in response, the minimum sample size necessary for toxicity evaluation was determined. The lens and vitreous of each living rabbit eye were examined using a slit lamp (SL-15, Kowa Co., Ltd.) after pupil dilatation with Mydrin-P (Santen Pharmaceutical Co., Ltd., Osaka, Japan), 4 h after injection (7.8, 78, and 780 nmol/eye) on day 1. This in-life examination was repeated twice on Days 2 and 7. Following this examination each day, the fundus was examined using an indirect ophthalmoscope (omega-500 brownbanded binocular ophthalmoscope; HEINE). On Day 8, animals were euthanized by exsanguination following anesthesia with xylazine hydrochloride (0.2 mL/kg, i.m.) and pentobarbital sodium (0.8 mL/kg, i.v.). The eyes were enucleated and fixed in a formalin-glutaraldehyde solution for 6–24 h, followed by a 10% neutral buffered formalin solution. Paraffin-embedded sections (1 mm thickness/each) were stained with hematoxylin and eosin and observed under a light microscope.

### In vitro assay for cell toxicity caused by vincristine

Human neuroblastoma cell line, SH-SY5Y (ECACC), was cultured in a flask containing a 1 to 1 mixture of Eagle minimum essential medium/Ham’s F12 medium (EMEM: F12) with 10% FBS and 1% penicillin/streptomycin (PS) at 37 °C in 5% CO_2_ and 3% O_2_ in air. When reached 70 to 80% confluency, cells were differentiated for three days in EMEM: F12 containing 2.5% FBS, 10 µM retinoic acid (RA), and 1% PS (Differentiation medium 1) and maintained for another three days in EMEM: F12 containing 1% FBS, 10 µM RA and 1% PS (Differentiation medium 2). The cells were then seeded and cultured for a day in a plate containing differentiation medium 2. They were further differentiated in neurobasal medium containing B27 supplement, Glutamax, 50 ng/mL BDNF, 1% PS, and 10 µM RA (Differentiation medium 3) for 9 days. Following a 16-day differentiation period, cells were treated with either vehicle (0.1% DMSO) or felodipine for 24 h and then treated concurrently with vincristine (200 nM) for another 24 h in differentiation medium 3. The cells were further maintained for three days in freshly prepared differentiation medium 3 containing vehicle or felodipine. Cells were observed under a light microscope, and phase-contrast images were taken periodically before and during drug treatment. After drug treatment, cell membrane and nuclei were stained with the cell membrane stain (Thermo Fisher Scientific, A14998), and hoechst33342 (Thermo Fisher Scientific, H3570), respectively, for 15 min at 37 °C. Three images per group were taken at 5x magnification using an Operetta CLS high-content analysis system (Perkin Elmer). Data are presented as the mean of three images. The number of nuclei and total neurite length were quantified and calculated using Harmony software (Perkin Elmer).

### In vivo determination of RGC survival in a rat ocular hypertensive model

The rats were randomly assigned to each group once they were enrolled in the study. The sample size was determined based on previous preliminary studies. As described in earlier studies^30,31^, all rats (except for the control group) received unilateral injection of 50 µL of hypertonic saline solution (1.85 M) into the episcleral vein of the left eye. Felodipine (4 µL, 40 nmol/eye) was intravitreally injected twice in the left eye of each rat in the treatment group on the day of OHT surgery (Day 1) and 11 days later. IOPs were measured using a Tonolab tonometer in both eyes before surgery (baseline) and on Day 1, Weeks 1, 2, and 3 after surgery. At each time point, IOP was measured 10 times consecutively, and these readings were averaged as the representative IOP. At week 3, the eyes were enucleated, perfused with 4% paraformaldehyde (PFA) overnight, and then stored in phosphate buffered saline (PBS).

To detect RGCs, RBPMS was used as a marker protein^32^ and whole-mounted retinas were immunostained with an anti-RBPMS antibody. The samples were washed in PBS and 0.5% Triton X-100 (Sigma-Aldrich, UK) and permeabilized by freezing at − 80 °C and thawing at room temperature alternately. They were blocked with 5% normal goat serum (Sigma-Aldrich, UK) in phosphate buffer (PB, 0.1 M) and incubated with guinea pig anti-RBPMS antibody at a dilution of 1:250 in bovine serum albumin (BSA) solution for two days. The resultant samples were washed in PBS and 0.5% Triton X-100 and further incubated with a secondary antibody solution (goat anti-guinea pig 647 nm, Alexa Fluor Invitrogen™ at 1:250 dilution in BSA solution). Samples underwent a final washing process and then were flat-mounted using Mowiol (Sigma-Aldrich, UK) and a coverslip (Merck, UK) onto a microscope slide. The flat-mounted retinas were observed under a fluorescent microscope (Olympus BX40, Windsor, UK with a 10 × Olympus lens; 1 pixel = 0.636 μm) with the 647 nm filter. When the rat eyes had unexpected retinal abnormality on histology dissection, they were excluded from the study.

### Statistical analysis

Statistical analyses were performed using EXSUS software version 10.0.7 and SAS version 9.4 (EPS Corporation, Tokyo, Japan) according to the manufacturer’s instructions. To compare the two groups, the F-test was used, followed by the Student’s *t*-test or Aspin-Welch test, whereas the paired t-test was used to compare pre- and post-treatment. For multiple comparisons, the Bartlett’s test was performed for equal variance, followed by the Dunnett’s test. Changes were considered statistically significant when the p-value was < 0.05.

## Electronic supplementary material

Below is the link to the electronic supplementary material.

Supplementary Material 1

## Data Availability

All data generated or analyzed during this study are included in this published article.

## References

[1] Weinreb, R. N. et al. Primary open-angle glaucoma. *Nat. Rev. Dis. Primers* 2 Preprint at (2016). 10.1038/nrdp.2016.67 10.1038/nrdp.2016.67 27654570

[2] Tham, Y. C. et al. Global prevalence of glaucoma and projections of glaucoma burden through 2040: A systematic review and meta-analysis. *Ophthalmology* **121**, 2081–2090 (2014). 24974815 10.1016/j.ophtha.2014.05.013

[3] Sharif, N. A. Elevated intraocular pressure and glaucomatous optic neuropathy: genes to disease mechanisms, therapeutic drugs, and gene therapies. *Pharmaceuticals* **16**, 870 (2023). 37375817 10.3390/ph16060870 PMC10303872

[4] Tribble, J. R. et al. Neuroprotection in glaucoma: mechanisms beyond intraocular pressure Lowering. *Mol. Asp Med.* **92**, Preprintathttpsdoiorg101016jmam2023101193 (2023). 10.1016/j.mam.2023.101193 37331129

[5] He, S., Stankowska, D. L., Ellis, D. Z., Krishnamoorthy, R. R. & Yorio, T. Targets of neuroprotection in glaucoma. *J. Ocul Pharmacol. Ther.* **34**, 85–106 (2018). 28820649 10.1089/jop.2017.0041 PMC5963639

[6] Sharif, N. A. Therapeutic drugs and devices for tackling ocular hypertension and glaucoma, and need for neuroprotection and cytoprotective therapies. *Front. Pharmacol*. **12**, 729249 (2021). 10.3389/fphar.2021.729249 10.3389/fphar.2021.729249 PMC8484316 34603044

[7] Mursch-Edlmayr, A. S., Bolz, M. & Strohmaier, C. Vascular aspects in glaucoma: From pathogenesis to therapeutic approaches. *Int. J. Mol. Sci.* 22 Preprint at (2021). 10.3390/ijms22094662 10.3390/ijms22094662 PMC8124477 33925045

[8] Alarcon-Martinez, L. et al. Neurovascular dysfunction in glaucoma. *Prog. Retin. Eye Res*. 97 Preprint at (2023). 10.1016/j.preteyeres.2023.101217 10.1016/j.preteyeres.2023.101217 37778617

[9] Araie, M. & Mayama, C. Use of calcium channel blockers for glaucoma. *Prog Retin Eye Res.* **30**, 54–71 (2011). 10.1016/j.preteyeres.2010.09.002 Preprint at. 20933604

[10] Wang, X. et al. The association between vascular abnormalities and glaucoma—what comes first? *Int. J. Mol. Sci.* **24**, Preprintathttpsdoiorg103390ijms241713211 (2023). 10.3390/ijms241713211 PMC10487550 37686017

[11] Alarcon-Martinez, L. et al. Interpericyte tunnelling nanotubes regulate neurovascular coupling. *Nature* **585**, 91–95 (2020). 32788726 10.1038/s41586-020-2589-x

[12] Hara, H., Toriu, N., Shimazawa, M. & Hara, H. Clinical potential of lomerizine, a Ca 2 + channel blocker as an anti-glaucoma drug: effects on ocular circulation and retinal neuronal damage. *Cardiovasc Drug Rev* **22**, 199–214 (2004). 10.1111/j.1527-3466.2004.tb00141.x 15492768

[13] Koseki, N. et al. A placebo-controlled 3-year study of a calcium blocker on visual field and ocular circulation in glaucoma with low-normal pressure. *Ophthalmology* **115**, 2049–2057 (2008). 18672290 10.1016/j.ophtha.2008.05.015

[14] Leung, G., Grant, A., Garas, A. N., Li, G. & Freeman, E. E. A systematic review and meta-analysis of systemic antihypertensive medications with intraocular pressure and glaucoma. *Am. J. Ophthalmol*. 255 7–17 Preprint at (2023). 10.1016/j.ajo.2023.03.014 10.1016/j.ajo.2023.03.014 36966883

[15] Vergroesen, J. E. et al. Association of systemic medication use with glaucoma and intraocular pressure: the European eye epidemiology consortium. *Ophthalmology* **130**, 893–906 (2023). 37150298 10.1016/j.ophtha.2023.05.001

[16] Lee, J. S. et al. Effect of antihypertensive medications on the risk of open-angle glaucoma. *Sci Rep* **13**, 16224 (2023). 10.1038/s41598-023-43420-3 PMC10533509 37758842

[17] Saltiel, E., Ellrodt, A. G., Monk, J. P. & Langley, M. S. Felodipine. A review of its pharmacodynamic and Pharmacokinetic properties, and therapeutic use in hypertension. *Drugs* **36**, 387–428 (1988). 3069435 10.2165/00003495-198836040-00002

[18] Johnson, J. D., Andrews, C. T., Khabbaza, E. J. & Mills, J. S. The interaction of felodipine with calcium-binding proteins. *J. Cardiovasc. Pharmacol.* **10** (Suppl 1), 53–59 (1987). 10.1097/00005344-198710001-00010 2442519

[19] Siddiqi, F. H. et al. Felodipine induces autophagy in mouse brains with pharmacokinetics amenable to repurposing. *Nat Commun* **10**, 1817 (2019). 10.1038/s41467-019-09494-2 PMC6472390 31000720

[20] Martinez, A. L. et al. Development of a novel in vitro assay to screen for neuroprotective drugs against iatrogenic neurite shortening. *PLoS One* **16**, e0248139 (2021). 10.1371/journal.pone.0248139 PMC7946280 33690613

[21] Swain, R. et al. Ocular delivery of felodipine for the management of intraocular pressure and inflammation: effect of film plasticizer and in vitro in vivo evaluation. *Int. J. Pharm.* **642**, 123153 (2023). 37339688 10.1016/j.ijpharm.2023.123153

[22] Sasaoka, M. et al. Intravitreal injection of endothelin-1 caused optic nerve damage following to ocular hypoperfusion in rabbits. *Exp. Eye Res.* **83**, 629–637 (2006). 16677631 10.1016/j.exer.2006.03.007

[23] Kawakami, M. B. K. T. N. Vasodilator actions of felodipine, a new Ca2 + entry blocker. *Nihon Yakurigaku Zasshi* **94**, 251–256 (1989). 10.1254/fpj.94.251 2613106

[24] Schocket, L. S., Grunwald, J. E. & Dupont, J. Effect of oral felodipine on ocular circulation. *Int. Ophthalmol.* **23**, 79–84 (1999). 10.1023/a:1026585515630 11196124

[25] Emre, M., Orgül, S., Haufschild, T., Shaw, S. G. & Flammer, J. Increased plasma endothelin-1 levels in patients with progressive open angle glaucoma. *Br. J. Ophthalmol.* **89**, 60–63 (2005). 15615748 10.1136/bjo.2004.046755 PMC1772488

[26] Marola, O. J., Syc-Mazurek, S. B., Howell, G. R. & Libby, R. T. Endothelin 1-induced retinal ganglion cell death is largely mediated by JUN activation. *Cell. Death Dis.* **11**, 811 (2020). 32980857 10.1038/s41419-020-02990-0 PMC7519907

[27] Sgambellone, S. et al. NCX 470 exerts retinal cell protection and enhances ophthalmic artery blood flow after ischemia/reperfusion injury of optic nerve head and retina. *Transl Vis. Sci. Technol.* **12**, 22 (2023). 37750744 10.1167/tvst.12.9.22 PMC10541723

[28] Taniguchi, T., Shimazawa, M., Sasaoka, M., Shimazaki, A. & Hara, H. Endothelin-1 impairs retrograde axonal transport and leads to axonal injury in rat optic nerve. *Current Neurovascular Research* **3**, 81–88 (2006). 10.2174/156720206776875867 16719791

[29] Akaishi, T., Kurashima, H., Odani-Kawabata, N., Ishida, N. & Nakamura, M. Effects of repeated administrations of tafluprost, latanoprost, and Travoprost on optic nerve head blood flow in conscious normal rabbits. *J. Ocul Pharmacol. Ther.* **26**, 181–186 (2010). 20334534 10.1089/jop.2009.0100

[30] Morrison, J. C. et al. A rat model of chronic pressure-induced optic nerve damage. *Exp. Eye Res.* **64**, 85–96 (1997). 9093024 10.1006/exer.1996.0184

[31] Husain, S. et al. PI3K/Akt pathway: A role in δ-opioid receptor-mediated RGC neuroprotection. *Invest. Ophthalmol. Vis. Sci.* **58**, 6489–6499 (2017). 29288267 10.1167/iovs.16-20673 PMC5749243

[32] Rodriguez, A. R., de Sevilla Müller, L. P. & Brecha, N. C. The RNA binding protein RBPMS is a selective marker of ganglion cells in the mammalian retina. *J. Comp. Neurol.* **522**, 1411–1443 (2014). 24318667 10.1002/cne.23521 PMC3959221

