# Supplementary material for: Felodipine re-positioned as a neuroprotectant via improved optic nerve head blood circulation in retinal ischemic rabbits and ocular hypertensive rats
Source: Sci Rep. 2025 Jul 3;15:23811. doi: 10.1038/s41598-025-09733-1 (PMC12229631; doi:10.1038/s41598-025-09733-1)
Supplement: Supplementary file 1 — Supplementary Material 1 [file 41598_2025_9733_MOESM1_ESM.pdf]

Supplementary Figure S1

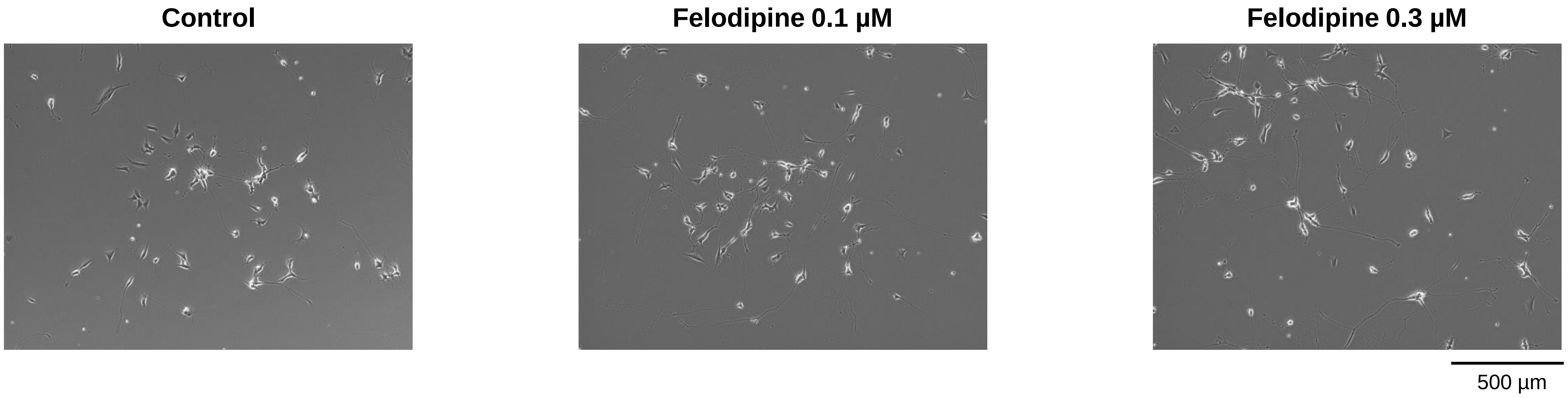

Supplementary Figure S1
Effects of felodipine on the cell morphology in differentiated SH-SY5Y cells.
Phase contract images were obtained after the pretreatment of felodipine (0.1 and 0.3 μM) for 24 hours in differentiated SH-SY5Y without vincristine.
